# Toxicity, Half-Life and Antitumor Activity of Phenyl 4-(2-Oxo-3-alkylimidazolidin-1-yl)benzenesulfonates as Novel Antimitotic CYP1A1-Targeted Prodrugs in Female Mouse Models

**DOI:** 10.3390/pharmaceutics17020233

**Published:** 2025-02-11

**Authors:** Atziri Corin Chavez Alvarez, Chahrazed Bouzriba, Vincent Ouellette, Mathieu Gagné-Boulet, Alexandre Patenaude, Sylvie Pilote, René C.-Gaudreault, Chantale Simard, Sébastien Fortin

**Affiliations:** 1 Faculté de Pharmacie, Université Laval, Québec, QC G1V 0A6, Canada; chahrazed.bouzriba.1@ulaval.ca (C.B.); vincent.ouellette.2@ulaval.ca (V.O.); mathieu.gagne-boulet.1@ulaval.ca (M.G.-B.); chantale.simard@pha.ulaval.ca (C.S.); 2 Centre de Recherche du CHU de Québec-Université Laval, Axe Oncologie, Hôpital Saint-François d’Assise, 10 Rue de l’Espinay, Québec, QC G1L 3L5, Canada; alexandre.patenaude@crchudequebec.ulaval.ca (A.P.); rene.c-gaudreault@crchudequebec.ulaval.ca (R.C.-G.); 3 Centre de Recherche de l’Institut Universitaire de Cardiologie et Pneumologie de Québec-Université Laval, Axe Cardiologie, Institut Universitaire de Cardiologie et de Pneumologie de Québec, 2725 Chemin Sainte-Foy, Québec, QC G1V 4G5, Canada; sylvie.pilote@criucpq.ulaval.ca; 4 Département de Médecine Moléculaire, Faculté de Médecine, Université Laval, Québec, QC G1V 0A6, Canada

**Keywords:** prodrugs, anticancer agents, antimitotics, CYP1A1, toxicity evaluation, half-life, antitumor activity

## Abstract

**Background/Objectives**: Chemoresistance of breast cancers (BCs) is a major impediment to current chemotherapeutics that urges the development of new drugs and new therapeutic approaches. To that end, phenyl 4-(2-oxo-3-alkylimidazolidin-1-yl)benzenesulfonates (PAIB-SOs) were recently prepared to fulfill some of the unmet needs with classic chemotherapeutics. PAIB-SOs are prodrugs bioactivated into potent antimitotics by the cytochrome P450 1A1 (CYP1A1), which is a frequent enzyme in resistant BC cells, but mostly missing in normal cells. Our screening program studies of PAIB-SO chemolibraries selected three prototypical PAIB-SOs as antimitotic prodrugs amenable for studies using BC animal models. **Methods**: Healthy female CD1^®^ IGS mice were treated with three prototypical PAIB-SOs, namely CEU-835, -934, and -938, for the determination of their toxicity and half-lives. Moreover, MCF7 tumor-bearing CD1-*Foxn1^nu^* Nude female mice were treated with the three prototypical PAIB-SOs for the determination of their antitumor activity. **Results**: Herein, we show that multi-intravenous administrations of CEU-835, -934, and -938 at their maximal solubilities are well tolerated in healthy female CD1^®^ IGS mice, as depicted by the evaluation of distress behaviors, organ necropsy, total blood cell count, and histology. Moreover, the half-life of CEU-835, -934, and -938 administered intravenously in healthy CD1^®^ IGS female mice were 8.1, 23.2, and 21.5 h, respectively. Finally, their intravenous administrations of CEU-934 and -938 decreased MCF7 tumor growth as efficiently as paclitaxel in MCF7 tumor-bearing CD1-*Foxn1^nu^* Nude mouse model. **Conclusions:** overall, our study demonstrated for the first time that pentyl-bearing PAIB-SOs are new CYP1A1-dependent prodrugs efficiently decrease breast cancer tumor growth, and show no side effects at their pharmacological concentration in mouse models.

## 1. Introduction

Breast cancers (BCs) are a major public health issue, despite significant progress in recent decades. They are the most common female cancers diagnosed, and the second cause of death from cancer among women worldwide [1]. BCs are a group of mammary gland diseases, notably characterized by abnormal cell proliferation, angiogenesis, and defective apoptosis signaling, which may induce metastasis at later stages [2]. Current therapies for BCs include tumor resection, mastectomy, radiation, immunotherapy, and various chemotherapies [3]. The latter include molecules such as the classic hormone therapy with tamoxifen and the chemotherapeutic paclitaxel. Unfortunately, the currently available anticancer armamentarium is unable to cure all forms of BC. As chemoresistance and metastasis emerge, more potent drugs exhibiting collaterally more side effects become required [4]. Therefore, the development of medicines precisely targeting these aggressive BC cells also exhibiting reduced deleterious effects in patients is vital [5].

**Figure 1:**
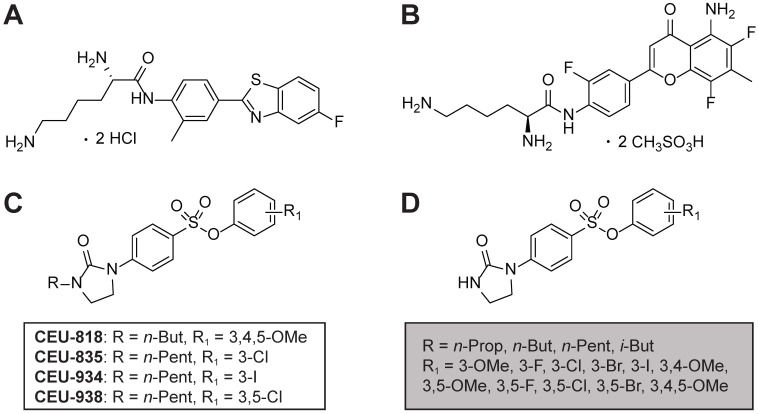
Molecular structures of (**A**) (2S)-2,6-diamino-*N*-(4-(5-fluorobenzo[d]thiazol-2-yl)-2-methylphenyl)hexanamide dihydrochloride (Phortress), (**B**) (2S)-2,6-diamino-*N*-(4-(5-amino-6,8-difluoro-7-methyl-4-oxochroman-2-yl)-2-fluorophenyl)hexanamide dimethanesulfonate (AFP464), (**C**) phenyl 4-(2-oxo-3-alkylimidazolidin-1-yl)benzenesulfonates (PAIB-SOs), and (**D**) phenyl 4-(2-oxoimidazolidin-1-yl)benzenesulfonates (PIB-SOs).

The use of rationally designed cytotoxic prodrugs provides a compelling approach to target cancer tumors without harming normal cells. Prodrugs are molecules exhibiting no or only modest pharmacological activities that are bioactivated either by enzymes and/or an appropriate chemical environment (e.g., acidic environment and hypoxia) [6]. The prodrug bioactivation into active metabolites by cancer cells reduces systemic side effects of antineoplastics, allowing higher doses of the drug to be administered safely. In this context, the detoxifying enzyme named cytochrome P450 1A1 (CYP1A1) has been confirmed as overexpressed in several cancers such as lung [7], digestive tract [8], and breast cancers [9]. CYP1A1 is modestly or unexpressed in healthy tissues such as lungs [10], digestive tract [11], and normal breast tissues [12], fostering the design of prodrugs based on the oxidative activity of that enzyme to kill selectively BC cells. To that end, (2S)-2,6-diamino-*N*-[4-(5-fluoro-2-benzothiazolyl)-2-methylphenyl]hexanamide dihydrochloride (Phortress, Figure 1A) [13] and (2S)-2,6-diamino-*N*-[4-(5-amino-6,8-difluoro-7-methyl-4-oxochroman-2-yl)-2-fluorophenyl]hexanamide dimethanesulfonate (AFP464, Figure 1B) [14] were prepared. The latter are CYP1A1-activated prodrugs of alkylating agents releasing selectively their alkylating metabolites within BC cells [15]. Unfortunately, clinical trials for Phortress and AFP464 were discontinued following unacceptable side effects [16].

Recently, we have developed a new generation of CYP1A1-activated prodrugs named phenyl 4-(2-oxo-3-alkylimidazolidin-1-yl)benzenesulfonates (PAIB-SOs, Figure 1C) generating antimitotic metabolites designated as phenyl 4-(2-oxoimidazolidin-1-yl)benzenesulfonates (PIB-SOs, Figure 1D) [17,18,19,20,21,22]. The mechanism of bioconversion of PAIB-SOs into PIB-SOs by CYP1A1 was confirmed, notably by a competition assay using known CYP1A1 inhibitors, their antiproliferative activity against cells transfected to overexpress CYP1A1, their apparent substrate affinity for CYP1A1, and their in vitro bioactivation in the presence of human CYP1A1 Supersomes™ [17,20,21]. It involves a C-hydroxylation mechanism on the alkyl side chain on the carbon atom adjacent to the nitrogen atom of the imidazolidin-2-one moiety of PAIB-SOs, followed by a spontaneous *N*-dealkylation of the carbinolamine-like intermediates [20]. In contrast to Phortress and AFP464, PAIB-SOs show antimicrotubule activity instead of alkylating activity [17,18,19,20,21]. Moreover, PAIB-SOs very slightly increase the expression of CY1A1 as compared to Phortress and AFP464 [17]. These key differences between their mechanism of action and their impact on CYP1A1 expression suggest that PAIB-SOs may also exhibit a completely different toxicity profile or pattern on normal tissues and organs than Phortress and AFP464. PAIB-SO derivatives exhibit in vitro antiproliferative activities in the nanomolar range. Likewise, the known antimitotics used to treat BCs block the cell cycle progression in the G2/M phase, inhibit the tubulin polymerization, and disrupt the microtubule dynamics, ultimately leading to cell death through apoptosis [17,18,19,20,21]. In the course of previous experiments, we identified the most potent PAIB-SO, namely 3,4,5-trimethoxyphenyl 4-(2-oxo-3-butylimidazolidin-1-yl)benzenesulfonate, as a lead compound (CEU-818, Figure 1C). Unfortunately, it showed a short retention time in mice (≤6 h), as well as a limited retention time in tumoral tissues (<6 h). To circumvent that impediment, we screened our chemolibraries of PAIB-SO to identify butyl- and pentyl-bearing PAIB-SOs as more stable toward human, mouse, and rat liver microsomes [19,20,21]. In the present study, three PAIB-SO derivatives, namely 3-chlorophenyl 4-(2-oxo-3-pentylimidazolidin-1-yl)benzenesulfonate (CEU-835), 3-iodophenyl 4-(2-oxo-3-pentylimidazolidin-1-yl)benzenesulfonate (CEU-934), and 3,5-dichlorophenyl 4-(2-oxo-3-pentylimidazolidin-1-yl)benzenesulfonate (CEU-938) (Figure 1C), were selected for in vivo studies, as they exhibited delayed biotransformation times in vitro with multi-species microsome assays [21]. Consequently, their side effects when administered multi-intravenously in healthy mouse models were evaluated. Afterwards, their half-lives were assessed in healthy mouse models and, finally, their antitumor activities in a MCF7 BC xenografted tumor mouse model were evaluated.

## 2. Materials and Methods

### 2.1. Reagents and Compounds

Paclitaxel (≥99.5%) Cat. No. AC328420050, sodium chloride powder Cat. No. S271-1, D-glucose Cat. No. D16-3, dimethyl sulfoxide Cat. No. AA22914K7, methanol HPLC grade Cat. No. A452SK-4, 2-propanol (isopropanol) HPLC grade Cat. No. A451SK-4, the ammonia solution 30% Cat. No. AC205840010, hydrochloric acid ACS Cat. No. AA33257P6, and ethanol anhydrous HPLC grade Cat. No. 6590-32 were purchased from Thermo Fisher Scientific (Waltham, MA, USA). Tetraglycol Cat. No. T3396, Cremophor^®^ EL Cat. No. 238470 and xylene Cat. No. MXX00604 were acquired from Millipore Sigma (St Louis, MO, USA). (±)-1-Methoxy-2-propanol (≥99.0%) Cat. No. CAAA41547-AK and Tween^®^ 80 Cat. No. 97061-674 were provided by VWR International, (Mont-Royal, QC, Canada). 17β-Estradiol pellets (0.36 mg; 90-day release) Cat. No. NE-121 and placebo pellets Cat. No. NC-111 were obtained from Innovative Research of America (Sarasota, FL, USA). CEU-835, -934, and -938 were prepared as described previously by Chavez Alvarez et al. [21]. The excipients used to solubilize PAIB-SOs were tetraglycol, ethanol, Tween^®^ 80, propylene glycol, glucose 10%, and NaCl 0.9% at 15, 1.60, 13.63, 5.45, 21.81, and 42.51% for CEU-835 and CEU-938 and at 15, 2.06, 17.65, 7.06, 28.23, and 30% for CEU-934, respectively. Paclitaxel was administered in a mixture containing 50% of NaCl 0.9% and 50% of a solution containing Cremophor^®^ EL, ethanol, and DMSO (49:49:2). Harris hematoxylin Cat. No. 3530-16 was provided by Ricca Chemical (Arlington, TX, USA); the 1% Alcoholic Eosin Y solution Cat. No. LC140302 was provided by LabChem™ (Zelienople, PA, USA).

### 2.2. Animals

CD1^®^ IGS female mice 16–18 g (https://www.criver.com/products-services/find-model/cd-1r-igs-mouse?region=24, accessed on 20 December 2024) and Nude CD-1 female mice CD1-*Foxn1^nu^* 18–23 g (https://www.criver.com/products-services/find-model/cd-1-nude-mouse?region=24, accessed on 20 December 2024) were purchased from Charles River Laboratories Canada (Laval, QC, Canada). Mice were kept under controlled temperature and humidity conditions with a controlled light and dark cycle, permanent water supply, and fed daily *ad libitum* with dry food. CD1^®^ IGS female mice were kept in a pathogen-controlled environment CD1-*Foxn1^nu^* Nude female mice in a pathogen-free environment with a filtered air environment. All animals were acclimated for seven days in animal facilities prior to the beginning of experiments.

### 2.3. Side-Effect Study of Multi-Intravenous Administrations of CEU-835, -934 and -938 in Healthy Mice

Animals were randomized into groups of five animals per cage. All of the animals were injected intravenously in the caudal vein biweekly (twice a week) for three weeks with a volume of 4 mL/kg for PAIB-SOs, vehicle or NaCl 0.9%. The NaCl 0.9% group was used to evaluate the effect of intravenous administration and animal handling, while the vehicle group was used to evaluate the effect of excipients. Each dose injected for CEU-835, -934, and -938 were 10, 15.6, and 3.6 mg/kg, respectively, corresponding to the maximum solubility of PAIB-SOs in the vehicle. The adverse effects were recorded for a 6 h period after each administration including abnormal movements, breathing, coordination, dermatitis, eye color, hydration, hyperactivity, inflammation, injuries, repetitive behavior, self-grooming activities, and skin color. The cumulative adverse effects were recorded before the end of the protocol, one week following the last administration, including all of the side effects listed above and the presence of masses, weight loss, and possible signs of pain. At the end of the protocol, animals were anesthetized with isoflurane, and they underwent a terminal cardiac puncture. Blood samples from three mice for each condition were preserved for evaluation of the total blood count. Then, still under anesthesia, the animals were euthanatized by decapitation, and a necropsy, as well as a macroscopic visual analysis of the liver, kidneys, and lungs were performed to assess the presence of necrosis and hemorrhages. Briefly, the thoracic cavity was opened and fixed over the animal’s head. The excess skin covering the peritoneum was resected for better visualization of the peritoneal cavity. The general appearance of the organs was evaluated macroscopically for the detection of necrotic tissue, hemorrhages, and other abnormalities in both the thoracic and the peritoneal cavities. Subsequently, the lungs, kidneys, and liver were analyzed ex vivo. The macroscopic analysis of organs was performed, including color, form, size, and presence or absence of necrotic tissues. Images of organs were recorded and then validated by the animal facilities coordinator. Blood was analyzed in triplicate for a total blood count by the Hematological service platform of Centre de Recherche du CHU de Québec-Université Laval. A one-way ANOVA was performed for the replicates in each experimental procedure in this section using the built-in option in the GraphPad Prism 9.3.1 software for statistical analysis. All errors are expressed as the standard deviation (Mean ± SD).

For the microscopic analysis for the toxicity evaluation, liver, kidneys and lungs were dissected from three mice in each treatment group treated with 0.9% NaCl, vehicle, CEU-835, CEU-934, or CEU-938. The organs were fixed in TISSUFIX^®^ (ChapTec, Montréal, QC, Canada) for two days, then washed with PBS and embedded in paraffin. The embedding process was conducted in a Tissue-Tek 1000 vacuum infiltration processor (Miles Scientific, Newark, DE, USA) using a temperature-controlled program at 38 °C. This included sequential 1 h baths in 50% ethanol, 80% ethanol, thrice of 100% ethanol, and thrice in toluene, followed by a final 3 h paraffin bath at 59 °C. After embedding, tissues were washed with toluene, 100% ethanol, and water. Paraffin blocks were formed using a Tissue-Tek paraffin dispensing console (Miles Scientific, Newark, DE, USA) and mounted in Histosette™ II cassettes (Thermo Fisher Scientific, Waltham, MA, USA). The paraffin blocks were cooled to −20 °C, sliced at 5 µm thickness using a Leica RM 2135 rotary microtome (Leica Microsystems Canada Inc., Richmond Hill, ON, Canada) with SHUR/Sharp™ disposable blades (Thermo Fisher Scientific, Waltham, MA, USA), and mounted on Superfrost™ Plus slides (Thermo Fisher Scientific, Waltham, MA, USA). For staining, slides were deparaffinized in toluene for 8 min, rehydrated in 100% ethanol for 2 min, followed by 95% ethanol for 1 min, and then stained with eosin-hematoxylin. The slides were washed in demineralized water for 2 min, and they were then incubated in filtered Harris hematoxylin for 35 min. The slides were rinsed in demineralized water for 1 min, immersed in 0.05% HCl for 1 min, and neutralized in 0.015% ammonia solution for 1 min. The slides immersed in 100% ethanol for 2 min, stained with alcoholic eosin (1%), and cleared with sequential baths of 100% ethanol (1 min), isopropanol (1 min), and xylene (2 min). The analysis of the samples was performed using an Olympus BX51 bright-field microscope (Olympus life science, Waltham, MA, USA). Images were acquired using a Q imaging RETIGA EXI digital camera (Q imaging, Surrey, BC, Canada) at 10× magnification and processed using the Image Pro Express software version 5.1.0.12 (Media Cybernetics, Rockville, MD, USA).

### 2.4. Half-Life Evaluations of CEU-835, -934 and -938 in Healthy Mice Following Intravenous Administration

CD1^®^ IGS female mice were randomized in groups of three animals for each condition studied. Animals were weighed prior the single intravenous administration in the caudal vein of either CEU-835, -934, or -938. Animals were anesthetized with isoflurane prior a terminal blood cardiac puncture at 0.5, 1, 2, 4, 8, or 12 h following the intravenous administration. Blood samples were collected in Microvette^®^ 500 EDTA K3E tubes (Sarstedt, Montréal, QC, Canada) and centrifuged at 1 × 10^3^ g for 15 min at 4 °C to isolate the plasma, which was stored at 4 °C until UHPLC analysis as described previously by Chavez Alvarez et al. [23]. Briefly, proteins of the plasma samples (40 µL) were precipitated using 500 µL of methanol. The resulting mixture was centrifuged for 5 min at 1.5 × 10^4^ g, the supernatant was separated from the pellet, and the pellet was discarded. The supernatant was filtered through a solid phase extraction column (SPE, IRIS™ MCX, 60 mg/3 mL, 25–35 µm cartridges, Canadian Life Sciences, Peterborough, ON, Canada) previously conditioned with 500 µL of methanol. The liquid was then collected, and 100 µL of DMSO was added to the sample prior evaporation using a SpeedVac^®^ Vacuum Concentrator (Thermo Fisher Scientific, Waltham, MA, USA) for 1.5–2 h. The samples were evaporated to a final volume of 50 µL. The samples were reconstituted in a final mixture of 20% DMSO, 40% methanol, and 40% water, and were analyzed by UHPLC-UV ACQUITY Arc system (Waters, Mississauga, ON, Canada) equipped with a 2998 UV/visible photodiode array detector, a CORTECS C18+ silica-based reversed-phase column (90 Å, 3.0 × 50 mm, 2.7 µm) paired with a CORTECS C18+ VanGuard Cartridge (90 Å, 2.1 × 5 mm, 2.7 µm, Waters, Mississauga, ON, Canada), and using a gradient of a water/methanol mixture. After the quantification of CEU-835, -934, or -938 in blood plasma by UHPLC-UV, half-lives were calculated using the half-life equation: half-life = 0.693/k, where k is the rate constant after plotting the concentration of each PAIB-SO at the analyzed times [24]. Each analyzed time point for every condition was repeated in triplicates, errors are expressed as the standard deviation.

### 2.5. Antitumoral Activity of CEU-835, -934, and -938 in an MCF7 Xenograft Tumor Mouse Model

CD1-*Foxn1^nu^* Nude female mice were randomized in groups of five animals per cage and underwent a subcutaneous administration of either a 17-ꞵ estradiol (0.36 mg) or a placebo pellet under isoflurane anesthesia in the left flank using 10G stainless steel trochars that have 90-day release capacities (Innovative Research of America, Sarasota, FL, USA). One week following the administration of the pellets, MCF7 (1 × 10^6^ cells) were injected subcutaneously under anesthesia with isoflurane in the right flank in a 100 µL mixture of MEM medium: Matrigel (1:1). Matrigel was prepared according to the manufacturer’s instructions (Corning, Montreal, QC, Canada). The tumor volumes were measured every other day with a caliper (Cat. No. S40515, Thermo Fisher Scientific, Waltham, MA, USA) until the tumors reached 50 to 100 mm^3^. Animals in groups of 8–10 individuals were then injected intravenously biweekly (twice a week; days 1, 4, 8, 11, 15, and 18) for three weeks in the caudal vein with: (1) NaCl 0.9%, (2) vehicle, (3) CEU-835 at 10 mg/kg, (4) CEU-934 at 15.6 mg/kg, or (5) CEU-938 at 3.6 mg/kg. Paclitaxel was injected once weekly at 50 mg/kg in the first week (day 1) and the following two weeks at 25 mg/kg (days 8 and 15). Animals were monitored for 6 h following each intravenous injection for any side effects/complications, such as stereotyped movement, skin color, hydration state, self-grooming, eye color, and general behavior. The weight of mice was recorded at least three times a week, and the tumor size was recorded at least once a week. A one-way ANOVA was performed for each set of data using the built-in option in the GraphPad Prism 9.3.1 software for statistical analysis. All errors are expressed as the standard deviation.

## 3. Results

### 3.1. Multi-Intravenous Administrations of CEU-835, -934, or -938 in Healthy Mice Exhibit No Behavioral and Macroscopic Side Effects

**Figure 2:**
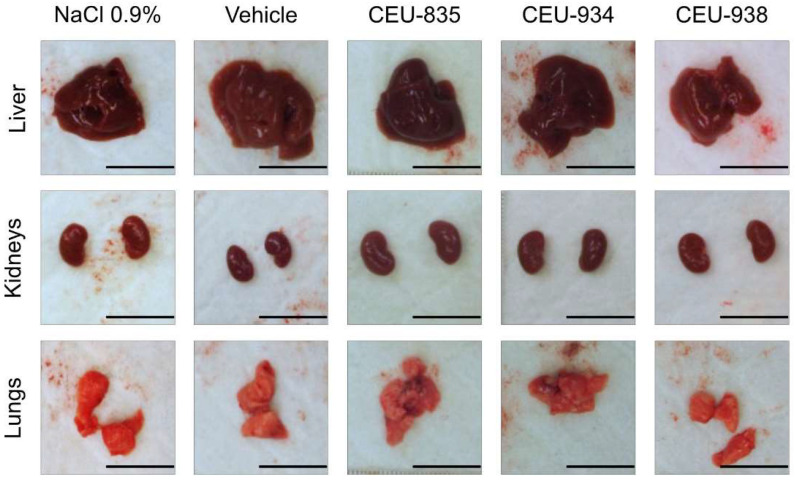
Representative macroscopic pictures of livers, kidneys, and lungs of CD1^®^ IGS female mice necropsied after multi-intravenous administration of NaCl 0.9%, vehicle, CEU-835, -934, and -938. The scale bar is set at 1000 mm. *n* = 3.

Firstly, we evaluated the innocuity of our PAIB-SOs CEU-835, -934, and -938 following multi-intravenous administrations using healthy CD1^®^ IGS female mouse models. PAIB-SOs were administered intravenously twice a week for three weeks at their maximal solubilities in the selected vehicle. For each administration, no sign of distress or discomfort were noticed 6 h post-administration. Moreover, behavior and observed appearance of mice after the vehicle and PAIB-SOs administrations were comparable to the normal saline group. All mice exhibited normal livers, kidneys, and lungs, with no macroscopic signs of necrotic tissue, as shown in Figure 2. No hemorrhage signs were present in animals, except for blood residues of the cardiac punctures in the vicinity of the heart for some animals. No sign of abnormal or augmented adipose tissue surrounding the organs was observed. The spleens were observed although they were not resected during the necropsy and their integrity including size, color and general appearance were evaluated as normal. The general observations of the necropsies of the animals that were treated with PAIB-SOs were macroscopically similar to the organs from the NaCl 0.9% and vehicle groups. Therefore, the multi-intravenous administration of PAIB-SOs at their maximal solubilities in vehicle does not show macroscopic side effects on healthy CD1^®^ IGS female mouse models.

### 3.2. CEU-835, -934, and -938 Show Normal Hematological Total Blood Count Profiles Following Their Multi-Intravenous Administrations in Healthy Mice

**Figure 3:**
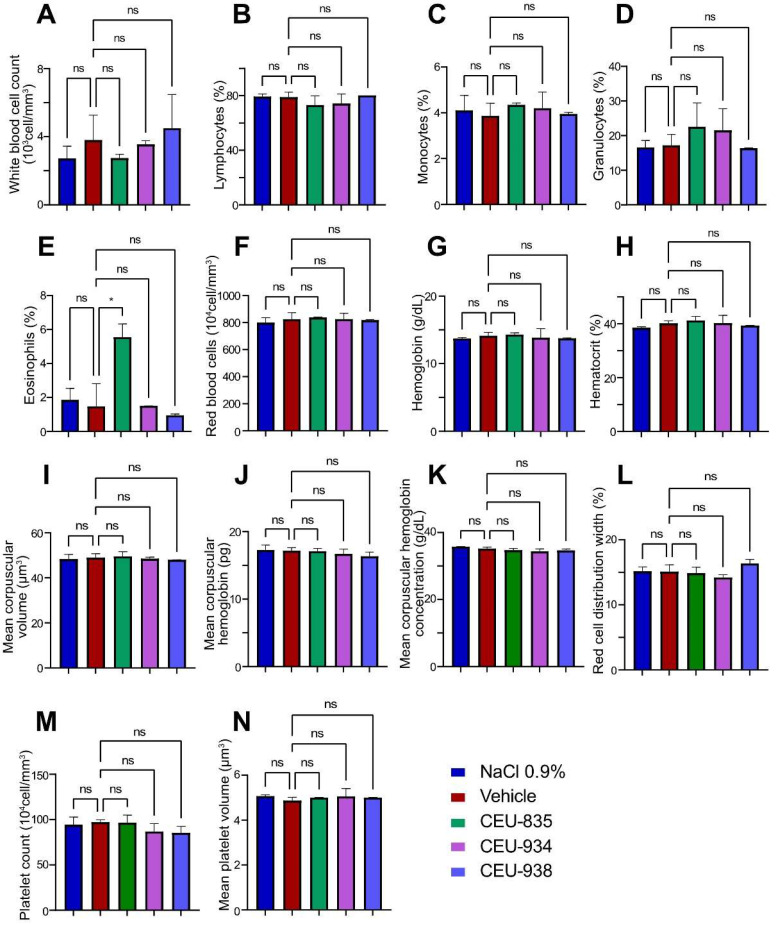
Total blood count profiles after multi-intravenous administration of NaCl 0.9% (*n* = 3), vehicle (*n* = 3), CEU-835 (*n* = 3), -934 (*n* = 3), and -938 (*n* = 3) in CD1^®^ IGS female mice of (**A**) white blood cells, (**B**) lymphocytes, (**C**) monocytes, (**D**) granulocytes, (**E**) eosinophils, (**F**) red blood cells, (**G**) hemoglobin, (**H**) hematocrit, (**I**) mean corpuscular volume, (**J**) mean corpuscular hemoglobin, (**K**) mean corpuscular hemoglobin concentration, (**L**) red cell distribution width, (**M**) platelet count, and (**N**) mean platelet volume. The *p*-value for all parameters is *p* < 0.05. ns: non-statistically significant. *: statistically significant.

To further study the innocuity of cumulative administrations of PAIB-SOs at the systemic level, we performed total blood count quantification one week after the last intravenous administration. First, our results pinpointed that after repetitive systemic administrations, PAIB-SOs did not stimulate inflammation nor cause immunodeficiency versus the control groups (saline and the vehicle), obtained for the different immune cells quantified including white blood cells (PAIB-SOs: 2.8–4.5 × 10^3^ vs. controls: 2.5–4.0 × 10^3^ cell/mm^3^, Figure 3A), lymphocytes (PAIB-SOs: 73–80% vs. controls: 78–80%, Figure 3B), monocytes (PAIB-SOs: 4.0–4.4% vs. controls: 3.6–4.1%, Figure 3C), granulocytes (PAIB-SOs: 16.4–23.0% vs. controls: 16.6–17.7%, Figure 3D) and eosinophils (PAIB-SOs: 1.0–5.6% vs. controls: 2.0–2.2%, Figure 3E). Nonetheless, CEU-835 induced a slight eosinophilia (5.6%) that is of statistical significance (*p* < 0.05) vs. the saline and vehicle groups. Second, the quantification of red blood cells and their derivatives after PAIB-SO regimens did not show any statistically significant difference as compared to the NaCl 0.9% and the vehicle groups (PAIB-SOs: 820–840 × 10^4^ vs. controls: 800–810 × 10^4^ cell/mm^3^, Figure 3F). As such, repeated systemic administrations of PAIB-SOs exhibited values of hemoglobin that were comparable to the control groups (PAIB-SOs: 13.8–14.3 vs. controls: 13.7–14.1 g/dL, Figure 3G), hematocrit (PAIB-SOs: 39.4–41.0% vs. controls: 38.3–40.0%, Figure 3H), mean corpuscular volume (PAIB-SOs: 48–50 vs. controls: 48–50 µm^3^, Figure 3I), mean corpuscular hemoglobin (PAIB-SOs: 16.0–17.0 vs. controls: 17.0–17.4 pg, Figure 3J), mean corpuscular hemoglobin concentration (PAIB-SOs: 34.0–34.8 vs. controls: 35.3–35.8 g/dL, Figure 3K), and red cell distribution width (PAIB-SOs: 15.0–16.0% vs. controls: 14.6–15.0%, Figure 3L). The last measures for the total blood count which concern platelets including platelet count (PAIB-SOs: 86–97 × 10^4^ vs. controls: 93–98 × 10^4^ cell/mm^3^, Figure 3M) and mean platelet volume (PAIB-SOs: 5.0–5.1 vs. controls: 4.9–5.0 µm^3^, Figure 3N) that did not exhibit any abnormality as compared to the control groups. In summary, with the exception of CEU-835 for the percentage of eosinophil cells, the multi-intravenous administrations of PAIB-SOs showed no statistically significant difference for all the analyzed parameters of hematological total blood count profiles as compared to control groups. Thus, it was concluded that no major apparent health issues were associated with the acute and subacute systemic administrations of CEU-835, -934, and -938.

### 3.3. The Microscopical Observation of Liver, Kidneys and Lungs of Healthy Mice Treated with CEU-835, -934 and -938 Show Normal Histological Morphology

**Figure 4:**
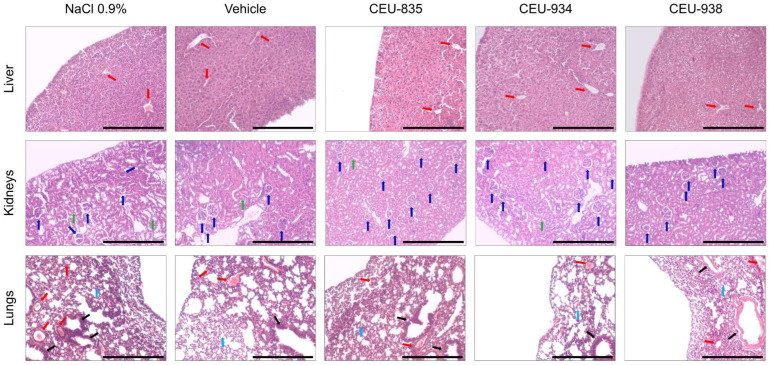
Representative pictures of the eosin-hematoxylin histological analysis at 10× of liver, kidneys, and lungs of CD1^®^ IGS female mice necropsied after multi-intravenous administration of NaCl 0.9%, vehicle, CEU-835, -934, and -938. The scale bar is set at 300 µm. The indicated tissue structures are blood vessels (red arrows), glomeruli (blue arrows), renal tubulointerstitium (green arrows), parenchyma (light blue arrows) and bronchoalveolar structures (black arrows). *n* = 3.

Histological analyses of dissected tissues were performed to detect microscopic anomalies or potential signs of toxicity derived from multi-intravenous administrations of CEU-835, -934, and -938 (Figure 4). Hematoxylin and eosin-stained liver sections from mice treated with 0.9% NaCl showed normal hepatic architecture, including well-defined blood vessels (red arrows), hepatocytes with abundant cytoplasm, and binucleated hepatocytes that are characteristic of this cell type. Liver sections from mice treated with either the vehicle, CEU-835, -934, or -938 were normal in appearance and similar to those treated with 0.9% NaCl. The analyzed kidney sections from CD1^®^ IGS female mice treated with 0.9% NaCl exhibit normal structural organization including a uniform distribution of glomeruli (blue arrows) and renal tubulointerstitium (green arrows), as well as normal cellular density. No difference in kidney tissue was observed between the groups treated with vehicle, CEU-835, -934, or -938 with that receiving 0.9% NaCl. Finally, stained lung sections from mice treated with 0.9% NaCl show normal distribution and density of parenchyma (light blue arrows), intact bronchoalveolar structures (black arrows) along with blood vessels with typical morphology (red arrows), and do not exhibit abnormal cellular infiltration. These results were also observed in treated groups when treated with either the vehicle, CEU-835, -934, or -938. Altogether, these results show that PAIB-SOs do not induce microscopic or pathological abnormalities in hepatic, renal, and pulmonary tissues.

### 3.4. CEU-835, -934, and -938 Have Systemic Half-Lives Ranging from 8.1 to 23.2 h

We then studied the systemic half-life of PAIB-SOs CEU-835, -934, or -938 following their single intravenous administration on healthy CD1^®^ IGS female mice. Our results show that CEU-835 exhibited the fastest systemic decay of the three molecules tested with a half-life of 8.1 ± 1.7 h. CEU-934 and -938 have comparable systemic decays, with half-lives of 23.2 ± 4.4 and 21.5 ± 1.7 h, respectively. Together, these results suggest that CEU-835, -934, and -938 systemic decays showing half-lives in mice over 8 h is significantly higher than CEU-818, and is favorable for further antitumor studies in mice at the dosage studied in the innocuity studies.

### 3.5. CEU-835, -934, and -938 Delayed or Decreased Tumor Growth of MCF7 BC Xenografted in Mice

**Figure 5:**
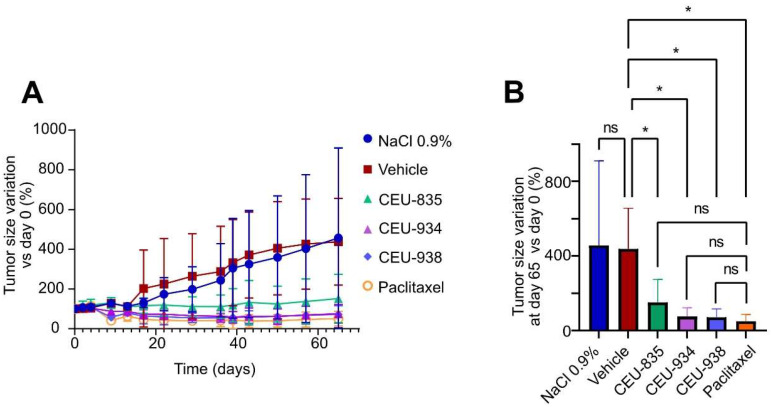
(**A**) Tumor growth curves expressing the tumor volume variation relative to the tumor volume at day 0. (**B**) Comparison of tumor volume variations at 65 days after the first intravenous administration compared to day-0 tumor volumes. The *p*-value for all parameters is *p* < 0.05. ns: non-statistically significant. *: statistically significant. *n* = 8–10.

As shown in our previous studies [17,20,21], PAIB-SOs exhibited antimitotic activity in several malignant BC cells that express CYP1A1 including MCF7. Therefore, the antitumoral activity of CEU-835, -934, and -938 was assessed using the murine MCF7 xenograft tumor model, which has been widely used for the assessment of drug antitumor activity for several decades [25,26]. In the course of the experiments, the three PAIB-SOs significantly reduced or arrested the growth of the tumors as compared to the saline or vehicle control groups (Figure 5A). At the end of the experiment, CEU-934 and -938 decreased the initial tumor volume by 25 and 29%, respectively, while CEU-835 limited tumor growth to 51% (Figure 5B). In contrast, saline and the vehicle groups show a similar increase in tumor growth at the endpoint of 357 and 338%, respectively. Finally, the paclitaxel used as a positive control showed a decrease in tumor volume by 50% as compared to the initial tumor volume, which is not statistically different from CEU-835, -934, and -938.

**Figure 6:**
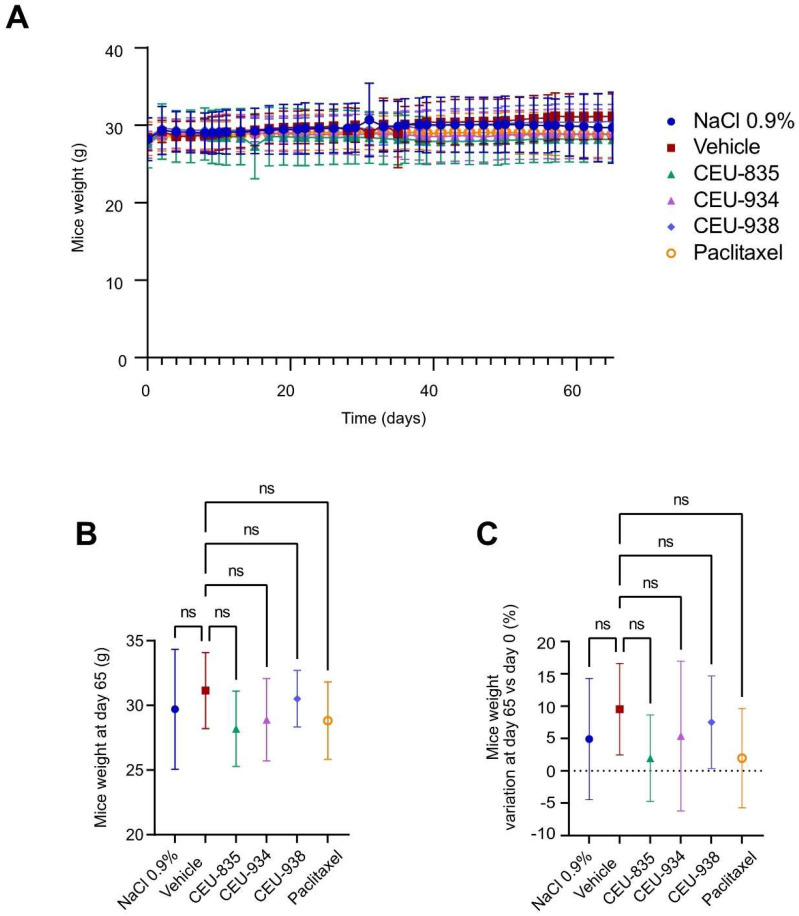
(**A**) Curves expressing the mice weight variation for 65 days after the first intravenous administration. (**B**) Mouse weights at day 65, and (**C**) curves expressing the mouse weight variations at day 65 vs. the mouse weights at day 0. The *p*-value is *p* < 0.05. ns: non-statistically significant. *n* = 8–10.

Moreover, mice showed no significant weight variations throughout the experiment, as well as no significant weight and weight percentage variations between the groups at the end of the experiment (Figure 6). Although no significant weight changes were observed in the paclitaxel group, the injection site was irritated, tender, and red for approximatively 24 h after the administration. Furthermore, the first administration of paclitaxel induced soft tissue damage on the mice tails, as well as a vesicant effect and impaired walking for several minutes. None of these side effects were observed for the other experimental groups. Altogether, these results showed that CEU-835, -934, and -938 have potent BC tumor growth inhibition properties, evidencing that their systemic administrations have no major impact on animal weight.

## 4. Discussion

BCs remain major health issues, despite the commercialization in recent years of new drugs including many targeted therapies. Therefore, there is still a high demand for novel therapeutic strategies for treating unresponsive BC patients. To that end, PAIB-SOs were developed to offer a new treatment alternative to BCs that respond poorly to current therapies or that develop chemoresistance. Moreover, PAIB-SOs are designed to reduce the side effects often found with classic antimitotics by targeting BC cells that express CYP1A1 limiting undesired outcomes in healthy CYP1A1-deficient cells and tissues.

In a previous screening program, a PAIB-SO referred to as CEU-818 was selected as the most potent [17,19,20,21]. However, its biodistribution and its excretion profiles in healthy OF1 female mice and in female nude mice bearing an MCF7 tumor using ^14^C-labeled molecule show a short retention time in mice (≤6 h) and a limited retention time in the tumor (<6 h) [21]. Moreover, our studies of the concentration–response relationship using different time periods evidenced that the antiproliferative activity of PAIB-SOs is concentration and time-dependent, and requires a contact time with BC cells between 24 and 36 h to achieve a significant cytocidal activity [19]. Therefore, to circumvent the suboptimal half-life of CEU-818 in mice that could limit its clinical development, we investigated the effect of the homologation of the alkyl side chain on the free nitrogen atom of the 2-imidazolidone moiety of PAIB-SOs. Our study showed that PAIB-SOs bearing an *n*-hexyl or an *n*-octyl groups were generally inactive at their maximum solubility in our vehicle, while PAIB-SOs bearing an *n*-pentyl group maintained an antiproliferative activity in the nanomolar range and a high selectivity ratio (1–3 logs) towards CYP1A1-expressing BC cells. Moreover, our study using mouse, rat, and human liver microsomes evidenced that the hepatic stability of PAIB-SOs is side chain length-dependent; the longer the side chain, the higher the metabolic stability. In addition, pentyl-bearing PAIB-SOs exhibited important antitumor activity on HT-1080 cells stably transfected with CYP1A1 and grafted onto the chorioallantoic membrane (CAM assay) of developing chick embryos and very low toxicity to chick embryos [21]. Therefore, we selected pentyl-bearing PAIB-SOs CEU-835, -934, and -938 for further preclinical studies based on (1) their promising half-lives in mouse, rat, and human liver microsomes, (2) their ability to not generate antimitotic metabolites in presence of hepatic enzymes, (3) their potent antiproliferative activity, (4) their selectivity towards BC cells, and (5) their solubility in the vehicles developed by our research group for intravenous administration. These encouraging properties prompted us to assess the safety profiles, half-life, and the antitumoral activity of selected pentyl-bearing PAIB-SOs on healthy and pathologic mouse models. First, we evidenced in this study that CEU-835, -934, and -938 can be administered safely in healthy mice without systemic and locoregional side effects. No distress behavior, and no abnormalities of thoracic and abdomen organs, nor major changes in blood count quantification, were detected following their repeated systemic administrations. Moreover, the blood count quantification is similar to the controls, and is considered normal according to sources that describe the correlation between total blood count abnormalities and diseases of health conditions in our murine models [27,28,29,30]. However, a slight eosinophilia was detected after CEU-835 administration. Nevertheless, none of the other parameters of the total blood count were altered in the CEU-835 group. Consequently, these results strongly suggest that this eosinophilia is not attributable to a specific immune reaction, to an infection, nor to any other clinical condition, as the animals did not show any other signs of side effects. In addition, the analyses of red blood cells and all their derivatives were found similar to the control groups, suggesting that the systemic administrations of selected pentyl-bearing PAIB-SOs do not cause dehydration, anemia, polycythemia abnormalities, and poikilocytosis defects. Additionally, no coagulation or bleeding problems were detected, since the platelet count and mean platelet volume were similar to the control groups. Finally, the histological analyses of the liver, kidneys, and lungs do not indicate any macroscopic abnormalities or toxicity associated with the administrations of PAIB-SOs. Based on these studies, we therefore concluded that no major apparent health issues or side effects were associated with the acute and the subacute systemic administrations of CEU-835, -934, and -938.

The second significant finding of our study is that the half-lives of PAIB-SOs were ≥8 h, which is significantly higher than that previously obtained with CEU-818. This increase in half-life is very supportive toward the design of a classic intravenous administration regimen and in line with our results obtained using mouse liver microsomes. Moreover, the half-lives (8.0–23.2 h) and the administered doses (3.6–15.6 mg/kg~135–400 µM in blood) are in agreement with the concentration and contact time with cancer cells required to achieve significant pharmacological activity. Thirdly, our results clearly show that CEU-835, -934, and -938 significantly delayed or decreased the growth of MCF7 tumors in the CD1-*Foxn1^nu^* Nude at a ratio comparable to paclitaxel, which is a gold standard treatment for resistant BCs unresponsive to first-line treatments [31]. Of note, we had to reduce the dose and the dosage of paclitaxel planned in the first week due to acute and subacute toxicity. Although further biodistribution and biotransformation analysis must be performed, our results strongly suggest that PAIB-SOs are substantially biodistributed within the MCF7 tumor tissue, and that they are bioactivated directly at the tumor site into their potent antimitotic PIB-SO metabolites.

Finally, our results are in line with the specific CYP1A1-dependent activation mechanism of PAIB-SOs, restricted to cancer cells expressing CYP1A1, and mostly absent in normal healthy tissues. Altogether, our study demonstrated the successful development of pentyl-bearing PAIB-SOs in mice, as new CYP1A1-dependent prodrugs for BC treatments that efficiently decrease BC tumor growth and show no side effects at their pharmacological concentration.

## Data Availability

All data are included in the manuscript.

## Abbreviations

The following abbreviations are used in this manuscript:

AFP464	(2S)-2,6-Diamino-*N*-[4-(5-amino-6,8-difluoro-7-methyl-4-oxochroman-2-yl)-2-fluorophenyl]hexanamide dimethanesulfonate
CEU-835	3-Chlorophenyl 4-(2-oxo-3-pentylimidazolidin-1-yl)benzenesulfonate
CEU-934	3-Iodophenyl 4-(2-oxo-3-pentylimidazolidin-1-yl)benzenesulfonate
CEU-938	3,5-Dichlorophenyl 4-(2-oxo-3-pentylimidazolidin-1-yl)benzenesulfonate
PAIB-SOs	Phenyl 4-(2-oxo-3-alkylimidazolidin-1-yl)benzenesulfonates
Phortress	(2S)-2,6-Diamino-*N*-[4-(5-fluoro-2-benzothiazolyl)-2-methylphenyl]hexanamide dihydrochloride
PIB-SOs	Phenyl 4-(2-oxoimidazolidin-1-yl)benzenesulfonates
